# Conserved Structure Associated with Different 3′CITEs Is Important for Translation of Umbraviruses

**DOI:** 10.3390/v15030638

**Published:** 2023-02-27

**Authors:** Sayanta Bera, Muhammad Ilyas, Anna A. Mikkelsen, Anne E. Simon

**Affiliations:** Department of Cell Biology and Molecular Genetics, University of Maryland, College Park, MD 20772, USA

**Keywords:** umbravirus, 3′CITE, BTE, cap-independent translation, SHAPE structure probing

## Abstract

The cap-independent translation of plus-strand RNA plant viruses frequently depends on 3′ structures to attract translation initiation factors that bind ribosomal subunits or bind directly to ribosomes. Umbraviruses are excellent models for studying 3′ cap-independent translation enhancers (3′CITEs), as umbraviruses can have different 3′CITEs in the central region of their lengthy 3′UTRs, and most also have a particular 3′CITE (the T-shaped structure or 3′TSS) near their 3′ ends. We discovered a novel hairpin just upstream of the centrally located (known or putative) 3′CITEs in all 14 umbraviruses. These CITE-associated structures (CASs) have conserved sequences in their apical loops and at the stem base and adjacent positions. In 11 umbraviruses, CASs are preceded by two small hairpins joined by a putative kissing loop interaction (KL). Converting the conserved 6-nt apical loop to a GNRA tetraloop in opium poppy mosaic virus (OPMV) and pea enation mosaic virus 2 (PEMV2) enhanced translation of genomic (g)RNA, but not subgenomic (sg)RNA reporter constructs, and significantly repressed virus accumulation in *Nicotiana benthamiana*. Other alterations throughout OPMV CAS also repressed virus accumulation and only enhanced sgRNA reporter translation, while mutations in the lower stem repressed gRNA reporter translation. Similar mutations in the PEMV2 CAS also repressed accumulation but did not significantly affect gRNA or sgRNA reporter translation, with the exception of deletion of the entire hairpin, which only reduced translation of the gRNA reporter. OPMV CAS mutations had little effect on the downstream BTE 3′CITE or upstream KL element, while PEMV2 CAS mutations significantly altered KL structures. These results introduce an additional element associated with different 3′CITEs that differentially affect the structure and translation of different umbraviruses.

## 1. Introduction

Unlike eukaryotic mRNAs that nearly invariantly contain a 5′ m7GpppN cap and a 3′ poly-A tail for efficient translation, plus-strand (+)RNA virus genomes translated in eukaryotic cells frequently require non-canonical mechanisms to outcompete cellular translation or to allow for translation when cap-dependent translation is suppressed [1,2]. Non-canonical translation frequently requires specific structures at the 5′ and/or 3′ ends of the genome to attract the translational machinery in the absence of a 5′ cap. For (+)RNA viruses infecting plants, two types of *cis*-structures/elements have been identified that support cap-independent translation [3]: internal ribosome entry sites (IRESs), which are more commonly found in animal viruses, and 3′ cap-independent translation enhancers (3′CITEs) [4,5]. Plant virus IRESs are normally located either near the 5′ end of the virus genome or proximal to internal ORFs and can either be large complex structures, as found in potyvirus triticum mosaic virus, or simple structures, as found in turnip mosaic virus [6,7,8].

3′CITEs are located either within the viral 3′UTR or can extend into the upstream ORF [4,5,9]. First identified in satellite tobacco necrosis virus [10], 3′CITEs facilitate viral RNA translation from the 5′ end of the genomic (g)RNA or subgenomic (sg)RNA by binding to eukaryotic translation initiation factors (eIFs) that subsequently recruit ribosomal subunits [11,12,13,14,15], or by binding directly to ribosomal subunits [16,17]. The initiation complex, which assembles at the 3′ end of the viral genome, must be transferred to the 5′ end, which is usually accomplished by a long-distance RNA:RNA interaction involving an unpaired sequence within the 3′CITE and an unpaired sequence at or near the 5′ end [4]. Using in vitro translation assays, the long-distance interaction was found to enhance the number of templates translated and not the rate of initiation or the number of ribosomes occupying the template [18]. Using high-throughput bi-cistronic translation assays, 3′CITEs have also been identified in human RNA viruses and cellular mRNAs, highlighting their conservation and importance [19].

Plant virus 3′CITEs have been organized into at least eight distinct classes based on conserved sequences and secondary structures and their ability to interact with specific translation factors [3,4,5]. Among the best-characterized 3′CITEs are the barley yellow dwarf virus-like translation element (BTE) [11,12,13,20], the panicum mosaic virus-like translational enhancer (PTE) [9,21,22], and the T-shaped structure (TSS) that was first identified in carmoviruses [23,24,25,26]. BTEs are characterized by a signature 17-nt motif that forms a short stem-loop (SL) implicated (with other sequences) in the binding of eukaryotic initiation factor (eIF)4G [11]. BTE s, which have been found in members of several genera within the *Tombusviridae*, were recently divided into three subclasses based on the number of apical SLs (at least two) and other conserved sub-class-specific features and motifs [20]. The PTE 3′CITE contains a Y-shaped secondary structure composed of a three-way junction with two upper SLs, a supporting stem of 6 to 7 bp, a large G-rich asymmetric loop, and a lower supporting stem of different sizes [9,21]. The G-rich sequence forms a critical pseudoknot (PK) with a C-rich sequence between the two SLs and is implicated in the binding of eIF4E [22]. PTEs were recently divided into two subclasses depending on the existence of an additional upstream hairpin that engages in a kissing loop interaction (KL) with the 3′ SL, an interaction that is required for the efficient formation of the PK [9]. Many PTEs (and BTEs) contain the motif 5′ UGCCA or its complement in the apical loop of one SL, which participates in the long-distance RNA:RNA interaction with a 5′ proximal hairpin apical loop or other unpaired sequences [4]. The PTE of pea enation mosaic virus 2 (PEMV2; umbravirus) is an exception by having a hairpin within an upstream 3′CITE (the adjacent “kissing loop T-shaped structure” [Kl-TSS]) engage in the long-distance interaction with the apical loop of a 5′ proximal, coding region hairpin [27]. The 3′TSS, found near the 3′ end of several carmoviruses and most umbraviruses, forms a tRNA-like structure [23,24,25] that directly interacts with ribosomes and 60S ribosomal subunits [16,27]. The connection between the 3′TSS and the 5′ end remains uncertain but has been proposed to involve bringing together 5′UTR-bound 40S subunits with 60S-bound 3′TSS [26]. Sequences, in addition to known 3′CITEs, have also been found to modulate translation. For example, tobacco necrosis virus D (TNV-D) and red clover necrotic mosaic virus RNA1 contain novel purine-rich sequences just upstream of their BTE 3′CITE, which are important for both virus accumulation and translation [28,29].

**Figure 1:**
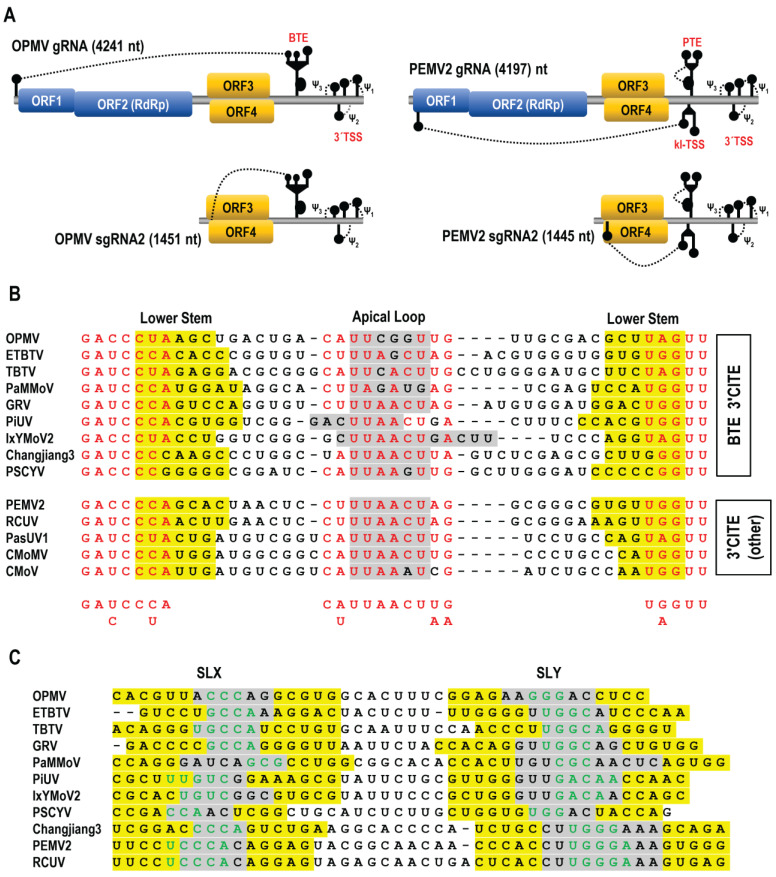
Novel conserved structures in the 3′UTRs of umbraviruses. (**A**). Umbravirus genome organization. ORFs encoding replication-required proteins are in blue, and movement proteins are in orange. A second, slightly larger sgRNA is present in some umbraviruses, including OPMV. 3′CITEs are shown along with the long-distance interaction (dashed line) connecting some 3′CITEs to the 5′ end. Other tertiary interactions near the 3′ end are also shown. (**B**). Sequence alignment in the CAS region. Conserved residues are in red. Residues in the CAS apical loop are shaded gray, and residues in the lower stem are shaded yellow. (**C**). Conserved structures just upstream of CAS in 11 umbraviruses. Two hairpins (SLX and SLY) are denoted with stem sequences in yellow and apical loops in gray. Putative kissing loop sequences are in green. OPMV, opium poppy mosaic virus; ETBTV, Ethiopian tobacco bushy top virus; TBTV, tobacco bushy top virus; PaMMoV, patrinia mild mottle virus; GRV, groundnut rosette virus; PiUV, picris umbravirus 1; IxYMoV2, ixeridium yellow mottle virus; Changjiang3, Changjiang tombus-like virus 3; PSCYV, paederia scandens chlorosis yellow virus; PEMV2, pea enation mosaic virus 2; RCUV, red clover umbravirus; PasUV1, Paederia scandens chlorosis yellow umbravirus; CMoMV; carrot mottle mimic virus; CMoV, carrot mottle virus.

Umbraviruses are unique within the *Tombusviridae* in having unusually long 3′UTRs (600 to 700 nt) containing several noncontiguous 3′CITEs in both the central region and proximal to the 3′ end [17,20,30]. Umbraviruses have a single (+)gRNA with four ORFs (Figure 1A); ORFs 1 and 2 are translated from the gRNA, and ORFs 3 and 4 are translated from a sgRNA [31,32]. ORF 1 codes for a replication-related protein, and ORF 2 encodes the RdRp, which is generated by a -1 ribosome frameshifting event near the end of ORF1 [33]. ORF 3 codes for an unstructured long-distance movement protein that also protects the sgRNA (and many cellular mRNAs) from nonsense-mediated decay [34]. ORF 4, which nearly completely overlaps with ORF 3, codes for a cell-to-cell movement protein in the 30K class of movement-related proteins [35]. For at least some umbraviruses, a second sgRNA is generated from the gRNA by a 5′ to 3′ exonuclease, which in opium poppy mosaic virus (OPMV) codes for an extension product of ORF 4 [20]. Umbraviruses do not encode a capsid protein or silencing suppressor, and while they can independently infect some hosts, they depend upon a helper virus for vector transmission [31].

Umbravirus PEMV2 has three 3′CITEs: a PTE and upstream adjacent Kl-TSS in the central portion of the 3′UTR; and a 3′TSS proximal to the 3′ end [17,27,36]. Using reporter constructs, the PEMV2 sgRNA was determined to require all three 3′CITEs for efficient translation in vivo, while gRNA constructs only required the Kl-TSS and PTE [30]. In contrast, OPMV contains two 3′CITEs, a centrally located BTE and the 3′ proximal TSS, with only the BTE used by both gRNA and sgRNA2 reporter constructs for translation in vivo [20].

The presence of the PEMV2 PTE/Kl-TSS and the OPMV BTE in similar internal locations within their 3′UTRs led us to examine if other umbraviruses have known CITEs or “CITE-like” structures in the same location. Here, we report that such structures were not only evident, but nearly all umbraviruses contain their known or putative 3′CITE atop stem scaffolds just downstream from a very similar small hairpin with an internal loop and conserved sequence at the base and in the apical loop. Virtually all mutations in this “CITE-associated structure” (CAS) in OPMV and PEMV2 significantly reduced virus accumulation in *Nicotiana benthamiana*, and many affected translation of cognate gRNA and sgRNA reporter constructs, supporting an important function for the element in translation. Mutations in CAS did not substantially affect the structure of the adjacent 3′CITE, but PEMV2 mutations significantly altered an upstream element conserved in 11 of 14 umbraviruses, including OPMV. These results identify the CAS as a new, centrally located 3′UTR structure just upstream of different types of 3′CITEs that is critical for the accumulation of umbraviruses and has differing effects on translation of their gRNA and sgRNA, suggesting the possibility that these umbravirus structures have evolved different functionalities.

## 2. Materials and Methods

### 2.1. Constructs

Plasmids pUC19-OPMV and pUC19-PEMV2 contain full-length OPMV and PEMV2 genomes, respectively, along with a T7 promoter upstream of the viral genome [20,27]. Similarly, in *Agrobacterium tumefaciens* binary vector pCB301, full-length OPMV and PEMV2 genomes were cloned downstream of the cauliflower mosaic virus 35S promoter and transformed into A. *tumefaciens*. Luciferase reporter constructs for OPMV gRNA (5′38 + U) and sgRNA2 (5′69 + U), and PEMV2 gRNA (5′89 + U) and sgRNA and (5′128 + U) were previously described [20,27]. Mutations in OPMV and PEMV2 constructs were introduced by QuikChange one-step site-directed mutagenesis [37] and were confirmed by sequencing (Eurofins Genomics).

### 2.2. In vitro Transcription of Full-Length Viral Genomes

Restriction enzymes, SmaI and PvuII (New England BioLabs), were used to linearize pUC19-OPMV and pUC19-PEMV2, respectively, and OPMV and PEMV2 luciferase reporter constructs were linearized with SspI, followed by in vitro transcription using T7 RNA polymerase. In vitro transcribed RNAs were purified by lithium chloride precipitation. The transcripts were quantified using a DeNovix DS-11 FX spectrophotometer, and the quality was assessed by ethidium bromide-stained agarose gel electrophoresis.

### 2.3. RNA Folding and SHAPE Modification of RNA

As previously described [9], RNA transcripts (12 pmol) were denatured at 65 °C for 3 min and then cooled on ice for 2 min. RNA was incubated in folding buffer [80 mM Tris–HCl (pH 8.0), 11 mM Mg(CH_3_COO)_2_, and 160 mM NH_4_Cl] for 10 min at 37 °C and then divided into two equal portions. A final concentration of 15 mM of N-methylisatoic anhydride (NMIA) dissolved in dimethyl sulfoxide (DMSO) was added to the (+) folded RNA, while an equal volume of DMSO was added to the folded (–) negative modification control. The (+) and (–) RNAs were incubated for 30 min at 37 °C, ethanol precipitated and then resuspended in 11 μL ddH_2_0 for reverse transcription.

### 2.4. Reverse Transcription of Modified RNA

Primers used for SHAPE probing were: OPMV_R1: 5′-GGATGGGAGTGACCACCAC (positions 4235–4217), OPMV_R2: 5′-CAGAACCCTCAGTTTGACTAC (positions 4008–3988), PEMV2_R1: 5′-AGGAAACAGCTATGACC (positions 4319 to 4302), PEMV2_R2: 5′-CGCGTTTGTGATCTTTTTGG (positions 3959 to 3940). For primer extension reactions, 2.5 µM of PET-labeled and 6-carboxyfluorescein (FAM)-labeled oligonucleotides were used with 6 pmoles of unmodified RNA (for sequencing reactions) or NMIA- and DMSO-treated RNA (sample reactions). SuperScript III reverse transcriptase (Invitrogen) was used for reverse transcription reactions as previously described [38,39]. To perform fragment analysis, cDNA products with ladders generated by Sanger sequencing were sent to Genewiz. Files related to fragment analyses were processed and analyzed using QuShape [40]. To verify that QuShape correctly subtracted the background from the corresponding peak positions, manual adjustments were implemented. RNA secondary structures were initially obtained using mFold [41] and modified by phylogenetic comparisons.

### 2.5. Agroinfiltration and RNA Gel Blot Analysis

OPMV and PEMV2 binary vector constructs were transformed into A. *tumefaciens* strain GV3101 and cultured in the presence of antibiotics. The cells were harvested and resuspended in resuspension buffer (10 mM MgCl_2_, pH 5.7, and 100 μM acetosyringone). For infiltrations of OPMV/PEMV2 WT and mutant constructs, an OD_600_ of 0.6 was mixed with the pothos latent virus p14-silencing suppressor construct [20] at an OD_600_ of 0.2 and infiltrated into two fully expanded leaves of *N. benthamiana*. After 72 h, agroinfiltrated leaves were harvested, and TRIzol (Invitrogen) was used to isolate the total RNA. For RNA gel blot analysis, the RNA was subjected to electrophoresis through 2% agarose gels and transferred to a charged nylon membrane by capillary action in 4× SSC (1× SSC is 0.15 M NaCl plus 0.015 M sodium citrate) buffer. After UV cross-linking, the membranes were hybridized with a mixture of three [α^−32^] dATP-labeled DNA oligonucleotides complementary to OPMV positions 3591 to 3630, 3634 to 3672, and 3676 to 3715. For PEMV2, a mixture of three [α^−32^] dATP-labeled DNA oligonucleotides complementary to positions 2731 to 2771, 2969 to 3004, and 3229 to 3270 were used. After incubation and washing, nylon membranes were exposed to a phosphorimager screen and scanned using an Amersham Typhoon Biomolecular Imager. The raw images were processed using ImageJ software to produce gel images and quantify band intensities.

### 2.6. Protoplast Transfection and In Vivo Luciferase Assays

Protoplasts prepared from callus cultures of *Arabidopsis thaliana* were transfected with luciferase reporter transcripts using a polyethylene glycol-mediated transformation protocol, as previously described [27]. Briefly, 5 × 10^6^ protoplasts were transfected with 20 μg of OPMV/PEMV2 luciferase reporter transcripts and incubated under constant light for 18 h at 22 °C. The cells were lysed at 18 h post-transfection, and the luciferase activity was assayed with a dual-luciferase reporter assay system (Promega) using a Modulus microplate multimode reader (Turner BioSystems). To check for the stability of the luciferase RNA constructs in protoplasts, the RNA gel blots were conducted using the total RNA extracted from protoplasts at 18 h post-transfection after exhaustive washing was performed.

### 2.7. RNA Structure Drawing

All of the RNA structures were generated using the on-line RNAcanvas web app (https://rna2drawer.app) accessed on 1 October 2022 [42].

## 3. Results

### 3.1. All Umbraviruses Contain a Conserved Hairpin in Their 3′ UTRs Located Upstream of Known or Putative 3′CITEs

**Figure 2:**
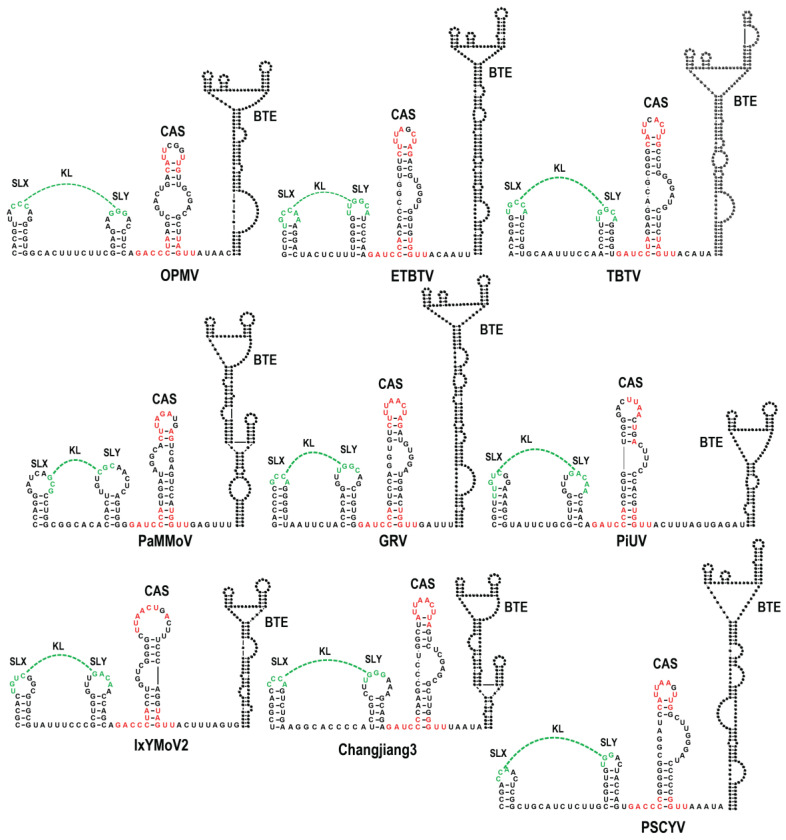
Predicted structures in the central region of umbraviruses containing BTE 3′CITEs. Conserved CAS sequences are in red. KL sequences are in green. Each BTE dot represents a nucleotide.

**Figure 3:**
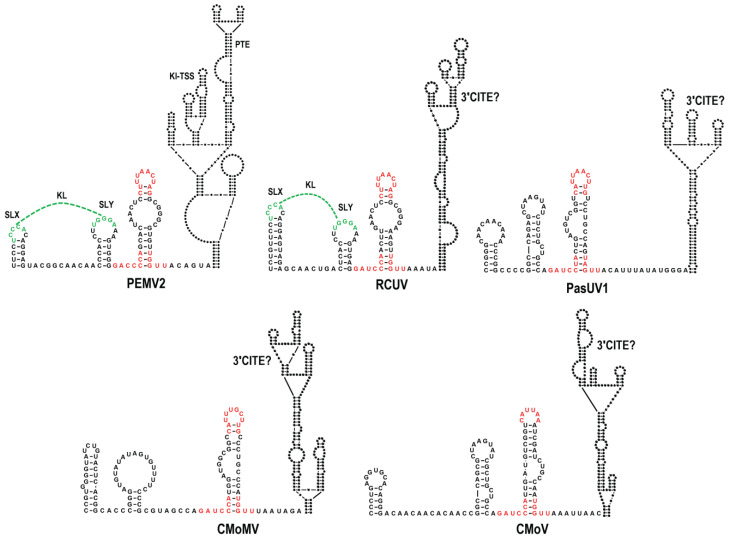
Predicted structures in the central region of umbraviruses containing non-BTE 3′CITEs. Conserved CAS sequences are in red. KL sequences are in green. Putative 3′CITEs are denoted by a question mark.

The sequences upstream of the internally located, previously defined 3′CITEs were aligned to identify any nearby conserved elements that might also play a role in translation. Just upstream (7 to 15 nt) from the base of a putative scaffold that supports known BTE 3′CITEs was a region with three discontinuous conserved motifs capable of folding into a small hairpin with an internal loop (Figure 1B top and Figure 2). All of the hairpins contained one of the conserved motifs fully or partially in the apical loop, with the other conserved sequences located at the base of the stem extending into flanking sequences on both sides. Analysis of the same region in non-BTE containing umbraviruses revealed that the same conserved sequences were present (Figure 1B, bottom), which for PEMV2 were similarly located just upstream from a putative scaffold containing its PTE and Kl-TSS 3′CITEs (Figure 3). The four umbraviruses without known 3′CITEs (PasUV1, CMoMV, RCUV, and CMoV) were predicted to have “CITE-like” structures atop scaffolds in identical locations downstream from the conserved hairpin (Figure 1B bottom and Figure 3). This positionally and partial sequence-conserved hairpin has been designated as the CITE-associated structure or CAS.

In addition, a second structurally conserved element was found to be 0 or 1 nt upstream of CAS in all BTE-containing umbraviruses and two non-BTE umbraviruses (PEMV2 and RCUV). This element consists of two similar-sized stem-loops (SLX and SLY) separated by 6 to 11 nt. SLX and SLY apical loops contain three to six residues capable of canonical base-pairing, thus forming a KL between the two stem-loops (Figure 1C, Figure 2, and Figure 3). For three umbraviruses, the KL included the motifs “GCCA” and “UGGC”, previously identified as common tertiary-interacting motifs in umbraviruses and carmoviruses [4,27]. SLX, SLY and the putative KL are referred to as the “KL element” in this report.

### 3.2. SHAPE RNA Structure Probing of OPMV gRNA and sgRNA CAS Region

**Figure 4:**
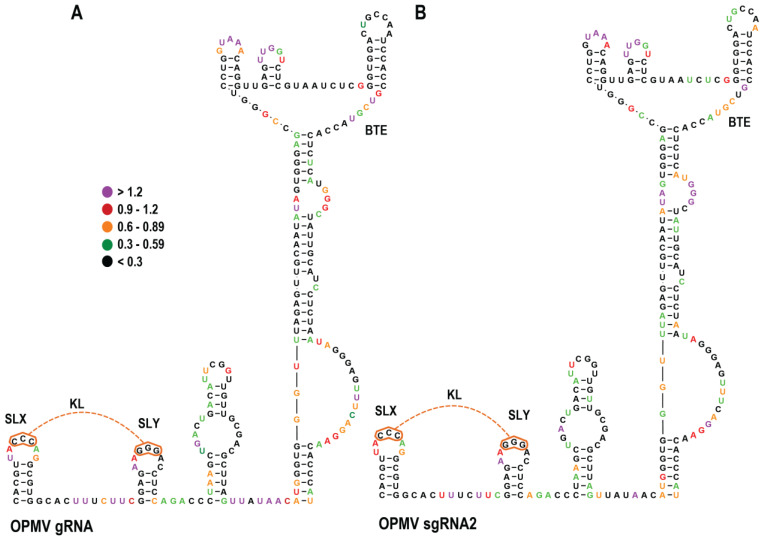
SHAPE RNA structure probing of the OPMV CAS region in full-length gRNA and sgRNA2 transcripts. (**A**). SHAPE was conducted using fluorescently labeled primers, which differed experimentally from previous SHAPE probing of the OPMV gRNA 3′UTR [20] but generated similar results. Individual residue “dots” are colored according to NMIA reactivity, from purple (most reactive) to black (least reactive). (**B**). SHAPE data for the CAS region in full-length OPMV sgRNA2 transcripts.

To investigate if the predicted CAS and KL element structures were present in OPMV transcripts, full-length gRNA and sgRNA2 transcripts were synthesized in vitro, denatured by heat and renatured by cooling, and then subjected to Selective 2′ Hydroxyl Acylation analyzed by Primer Extension (SHAPE) RNA structure probing (Figure 4). SHAPE reports on the flexibility of residues, with unpaired residues usually being more flexible and thus more capable of assuming a conformation necessary for interaction with SHAPE reagents [38]. SHAPE reactivities for both OPMV gRNA and sgRNA2 residues were similar in the CAS and BTE region and consistent with the presence of SLX, SLY, and KL. However, the lower CAS stems in both gRNA and sgRNA2 transcripts were not supported by SHAPE data, as both contained similar SHAPE-reactive residues on the 5′ side (Figure 4). In contrast, the BTE structures for both gRNA and sgRNA were well supported by SHAPE data, as was previously shown for the gRNA [20], with similar SHAPE-reactive residues mainly in unpaired regions. One prominent exception was the base of the BTE scaffold, with three residues on the 5′ side and two residues on the 3′ side moderately reactive with the SHAPE reagent.

### 3.3. OPMV CAS Is Important for Viral Accumulation In Vivo

**Figure 5:**
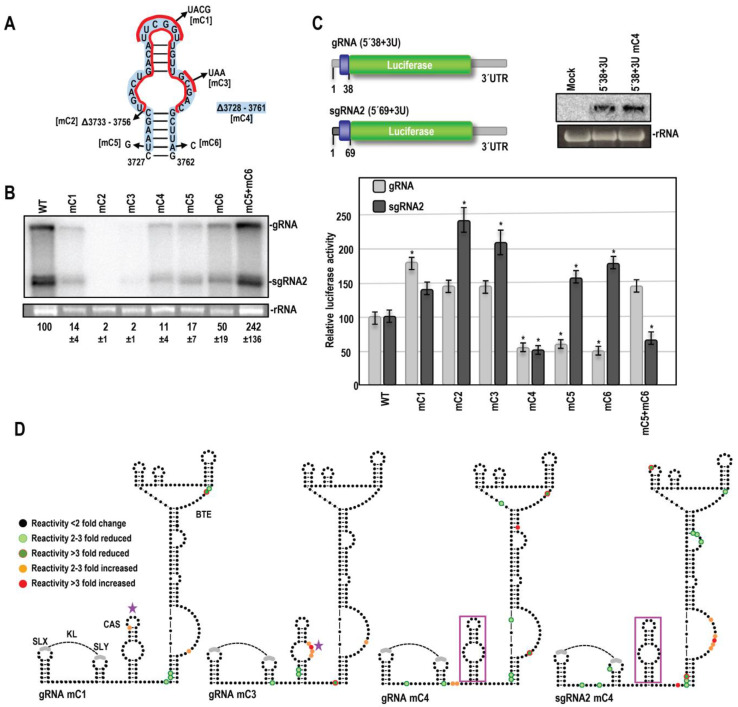
CAS is important for OPMV accumulation in *N. benthamiana* and efficient translation of reporter constructs. (**A**). CAS nucleotide alterations and deletions. Names of mutations are in brackets. Red lines help define some of the mutations and deletions. Blue shading denotes residues deleted in mC4. (**B**). RNA gel blot analysis of total RNA extracted from systemically infected *N. benthamiana* following agroinfiltration with either WT or mutant OPMV. Ethidium bromide-stained 26S rRNA was used as the loading control, with which band intensities were normalized. Standard error is shown for three independent experiments. (**C**). Relative translation of OPMV gRNA and sgRNA reporter transcripts containing full-length 3′UTR and 5′ terminal sequences [non-coding (gray) and coding (blue)] necessary for efficient translation as previously described [20]. gRNA construct; 5′ terminal 38 nt (5′38 + U). sgRNA2 construct; 5′ terminal 69 nt (5′69 + U). Luciferase activity was assayed 18 h following protoplast transfection. Error bars denote standard deviation for three independent assays. ‘*’ Denotes statistical difference with *p* < 0.05 analyzed using the *t*-test. Right panel shows approximately equal accumulation of gRNA parental and mC4 luciferase transcripts at the 18 h time point as assayed by RNA gel blots, indicating that the deletion was not affecting the stability of the transcripts. Ethidium bromide-stained 26S rRNA was used as a loading control. (**D**). SHAPE RNA structure probing of selected OPMV gRNA and sgRNA full-length CAS mutant transcripts. Colors denote changes in mutant nucleotide reactivity relative to WT. Black dots, <2-fold change; light green, 2- to 3-fold reduction; dark green; >3-fold reduction; orange, 2- to 3-fold increase; red, >3-fold increase. Star denotes location of alterations. CAS deletion is boxed in magenta. SHAPE data were averaged from at least three independent replicates.

To test for the importance of the phylogenetically conserved OPMV CAS structure, deletions and base modifications were introduced into base-paired, apical, and internal bulge loops (Figure 5A). WT and mutant gRNA accumulation were assessed using RNA gel blots following the *Agrobacterium tumefaciens*-mediated infiltration of expression constructs into *N. benthamiana*. The conversion of the conserved CAS 6 nt apical loop into a UNCG-type tetraloop (mC1) decreased virus accumulation by 7-fold (Figure 5B). Deletion of the upper portion of CAS, including the internal bulge (mC2), or alteration of three residues on the 3′ side of the bulge (mC3) reduced virus accumulation to near undetectable levels. Surprisingly, the deletion of the entire CAS (mC4) was not as detrimental, with the gRNA reaching 11% of WT levels. To address the presence of the CAS hairpin structure, single and compensatory mutations were introduced into the lower stem. Single mutations mC5 and mC6 were both detrimental, reducing virus accumulation to 17 and 50% of WT levels, respectively. mC5 + mC6, which should reestablish the lower stem, restored virus accumulation to at least WT levels. Altogether, these results strongly support the phylogenetically conserved CAS structure and the importance of the element for the efficient accumulation of OPMV in *N. benthamiana*.

### 3.4. OPMV CAS Is Required for Efficient Translation of gRNA and sgRNA2 Reporter Constructs In Vivo

The conserved location of CAS hairpins just upstream of known or putative 3′CITEs in all umbraviruses suggested a possible conserved function in translation mediated by the adjacent 3′CITE. To begin testing this hypothesis, CAS deletions and base alterations were introduced into firefly luciferase reporter constructs previously used to analyze sequences involved in translation of the gRNA and sgRNA2 [20] (Figure 5C). Both constructs contain the full-length 3′UTR along with 5′ sequences sufficient for efficient translation, which includes the BTE long-distance interacting motif. This required that the gRNA construct include the gRNA 5′ terminal 38 nt (5′38 + U) and the sgRNA2 construct include the sgRNA 5′ terminal 69 nt (5′69 + U). The transcripts were transformed into *A. thaliana* protoplasts and luciferase activity was measured 18 h later. Replacing the CAS apical loop with a UACG tetraloop (mC1) increased 5′38 + U luciferase activity by 1.8-fold but did not significantly affect translation of 5′69 + U (Figure 5C). The removal of the top half of the CAS (mC2) or mutations in the bulge (mC3) enhanced translation of 5′38 + U by nearly 50% (although this increase was not considered significant). Translation of 5′69 + U increased by 2.1- and 2.4-fold, respectively. In contrast, the complete removal of CAS (mC4) caused a two-fold decrease in translation of both 5′38 + U and 5′69 + U. The transcript levels remaining at the 18-h time point did not differ for 5′38 + U and 5′38 + U mC4, indicating that the deletion was not affecting the stability of the input RNA (Figure 5C, right). The disruption of the lower CAS stem (mC5 and mC6) had contrasting effects on the gRNA and sgRNA constructs, decreasing luciferase activity by nearly 2-fold in 5′38 + U while increasing luciferase activity by 1.6- and 1.8- fold in 5′69 + U. Reestablishing the lower stem (mC5 + mC6) reversed the effects of the single mutations, increasing translation of 5′38 + U to a level greater than WT and decreasing translation of 5′69 + U. These results support an important but differential role for CAS in translation of the gRNA and sgRNA constructs associated with specific 5′-proximal sequences, the only difference between the two constructs.

### 3.5. Effect of OPMV CAS Mutations on BTE and KL Structures

To address how CAS might be impacting translation and assess its direct or indirect connection (if any) with the upstream KL element and downstream BTE, full-length OPMV gRNA transcripts containing a subset of the CAS mutations were subjected to SHAPE structure probing (Figure 5D). The data are color coded for individual residues, with orange and red representing SHAPE reactivity increases of 2–3 fold or >3-fold, respectively, and light green and dark green representing reactivity decreases of 2–3 fold or >3-fold, respectively.

Altering the CAS apical loop (mC1) did not significantly change residue reactivity in the KL element (i.e., less than a 2-fold change), suggesting that the KL element is not interacting with the conserved CAS apical loop. In contrast, residues on the 5′ side of the BTE scaffold lower stem that were normally reactive by SHAPE (see Figure 4A) had significantly reduced reactivity, as did two residues in the upper portion of the BTE that are part of a conserved BTE motif [20]. The remainder of the BTE was virtually unchanged, suggesting that the conserved apical loop did not interact directly with the 3′CITE.

CAS interior bulge mutation (mC3) caused a substantial increase in the reactivity of the residues at the mutation site, suggesting that this bulge sequence is paired in the WT gRNA transcript (Figure 5D). Additionally, residues that were normally SHAPE reactive in the CAS lower stem showed a significant reduction in reactivity, suggesting that CAS was now assuming its phylogenetically conserved hairpin structure. The single reactive residue in the stem of SLY in WT transcripts was also significantly less reactive, suggesting stabilization of the SLY stem-loop. Only a single BTE residue (in the large bulge loop near the base) showed enhanced reactivity, suggesting that the WT CAS bulge loop sequence was not interacting with the BTE.

Deletion of the entire CAS hairpin (mC4) caused similar reductions in reactivity in the BTE lower stem and upper conserved motif as mC1 (Figure 5D). Four additional scattered residues in the BTE displayed reactivity changes due to the CAS deletion. The single residue in SLY that showed reactivity reduction for mC3 was similarly reduced in mC4 transcripts, suggesting a similar consequence associated with the loss of these CAS bulge loop sequences.

When sgRNA2 transcripts with the CAS deletion (mC4) were subjected to SHAPE structure probing, both similar and unique changes in residue reactivity were evident. In the BTE, very similar reactivity reductions were found at the base of the BTE and in the upper conserved motif. Two additional bulge loop regions in the BTE were uniquely altered: the upper bulge (and closing base-pair) had three of seven residues with reduced reactivity while the large bulge near the BTE base had four residues with enhanced reactivity. In the KL element, one residue in the loops of the SLX and SLY hairpins had reduced reactivity, along with two residues between the two hairpins. Since gRNA and sgRNA2 share identical sequences in this location, these results suggest that this location does not constitute a structurally separate domain in gRNA and sgRNA2 transcripts but is interacting with sequences in the gRNA that are missing in the sgRNA2 or that assume a different conformation.

### 3.6. Examination of Conserved CAS Flanking Sequence and a Complementary Sequence in the BTE 17-nt Signature Motif

**Figure 6:**
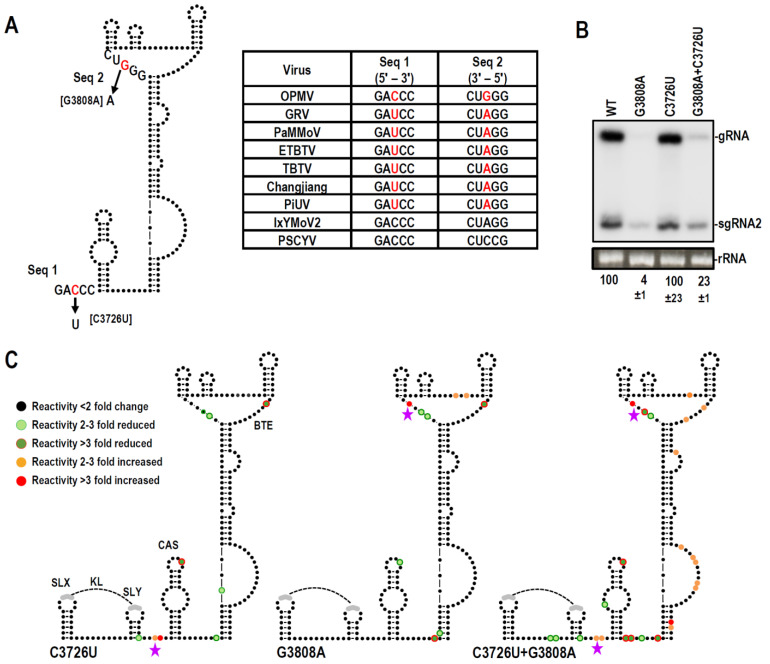
Direct interaction between CAS- and BTE-conserved sequences is not supported by SHAPE. (**A**). Location of the alterations and table showing conserved sequence just upstream of CAS that can potentially pair with conserved sequence in the BTE. OPMV contains a covariant change not present in other umbraviruses. (**B**). RNA gel blot analysis of total RNA extracted from systemically infected *N. benthamiana* following agroinfiltration with either WT OPMV or CAS/BTE mutants. Ethidium bromide-stained 26S rRNA was used as a loading control. Standard error is shown from three independent experiments. (**C**). SHAPE probing of OPMV gRNA mutants. Colors represent changes in nucleotides reactivity relative to WT. See legend to Figure 5. Star indicates location of alterations. SHAPE data were averaged from at least 3 independent replicates.

As shown in Figure 1B, Figure 2 and Figure 3, sequences flanking both sides of the CAS are strongly conserved in all umbraviruses. Curiously, the upstream flanking sequence, 5′GA C/U CC, is complementary to the sequence at the 5′ end of the 17-nt conserved motif in seven of nine umbravirus BTEs (5′GG G/A UCCUGGUAAACAGG), including a covariant change in OPMV when compared with other umbraviruses (Figure 6A). To determine if there is a direct interaction between the CAS and BTE mediated by these sequences, single mutations were individually generated in the two motifs, converting them into the same sequence found in the majority of BTE-containing umbraviruses (5′GACCC:GGGUC to 5′GAUCC:GGAUC). The BTE mutation G3808A, which should disrupt the putative interaction while maintaining a consensus 17-nt BTE signature sequence, reduced OPMV accumulation in *N. benthamiana* to near undetectable levels (Figure 6B). In contrast, the corresponding mutation upstream of the CAS (C3726U), which maintains the conserved consensus sequence while disrupting the putative CAS-BTE interaction, had no effect on OPMV accumulation in *N. benthamiana*. When present together in the same transcript, OPMV levels were enhanced nearly six-fold compared with G3808A alone, suggesting a possible slight compensatory effect. However, the mutant OPMV still accumulated poorly (23% of WT) despite containing the more conserved versions of the two motifs.

Since mutagenesis assays did not conclusively rule in (or out) an interaction between the two conserved motifs, transcripts containing the single and double mutations were subjected to SHAPE structure probing. Our reasoning was that disruption of an interaction between the two motifs should cause structural changes in the partner sequence that might be reduced by compensatory mutations. C3726U caused enhanced reactivity at the mutation site upstream of CAS, suggesting that this sequence is normally paired (Figure 6C). However, no reactivity changes were evident in the proposed interacting BTE motif, although several other BTE residues showed reduced reactivity. BTE mutation G3808A both enhanced and reduced the reactivity of residues at the mutation site and at several other sites in the BTE but not in the corresponding motif upstream of CAS. Transcripts containing both C3762U and G3808A showed an additive effect of the changes due to the individual mutations, as well as a substantial number of additional reactivity changes in the BTE and other sequences in the region (Figure 6C). Therefore, the SHAPE data do not support a direct interaction between the two conserved motifs in the initial structure assumed by these transcripts.

### 3.7. Disrupting the KL between SLX and SLY Does Not Affect Accumulation in N. benthamiana

**Figure 7:**
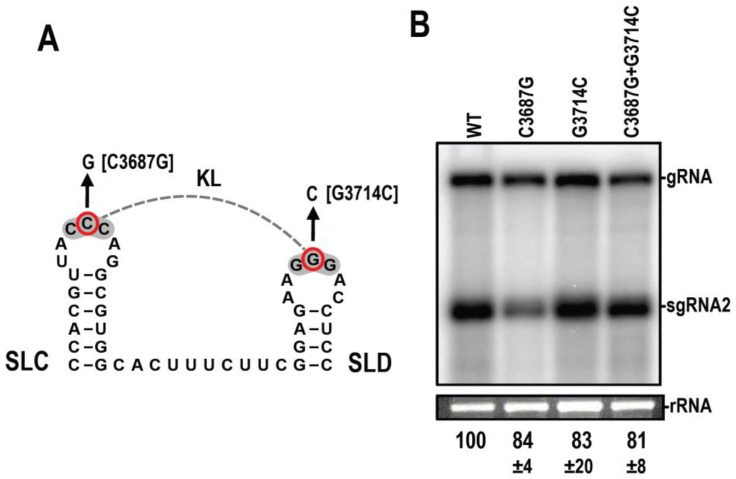
Disruption of the putative KL between SLX and SLY does not affect the accumulation of OPMV in infiltrated leaves. (**A**). C3687G and G3714C (indicated by red circles) were introduced to disrupt a KL. (**B**). RNA gel blot analysis of total RNA extracted from systemically infected *N. benthamiana* following agroinfiltration with either WT OPMV or OPMV containing either single or double mutations. Ethidium bromide-stained 26S rRNA was used as a loading control.

Eleven of fourteen umbraviruses have a similarly positioned KL element (Figure 1C, Figure 2 and Figure 3). To test for the presence and importance of the KL interaction in the accumulation of OPMV in *N. benthamiana*, single and compensatory mutations were generated, and the levels were determined by RNA gel blots (Figure 7). gRNAs containing single and double mutations accumulated between 81 and 84% of WT OPMV, a decrease that was not significant. The purpose of this putative KL interaction conserved in the majority of umbraviruses thus remains unknown.

### 3.8. SHAPE RNA Structure Probing of the PEMV2 gRNA and sgRNA3′UTR

**Figure 8:**
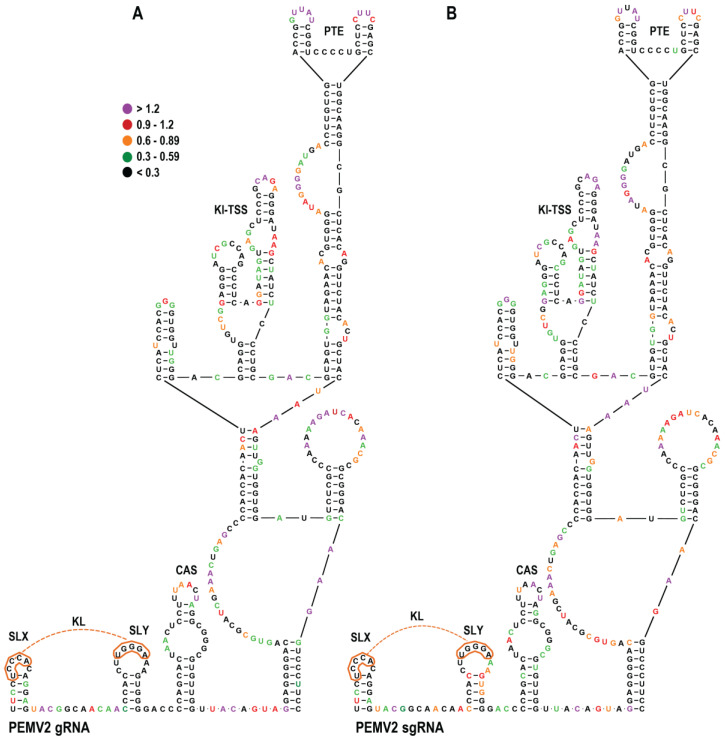
SHAPE probing of the CAS region in full-length PEMV2 gRNA and sgRNA. (**A**). SHAPE reactivity of gRNA residues. Individual residue “dots” are colored according to NMIA reactivity, from purple (most reactive) to black (least reactive). gRNA structures are labeled for clarity. (**B**). SHAPE reactivity of sgRNA residues.

**Figure 9:**
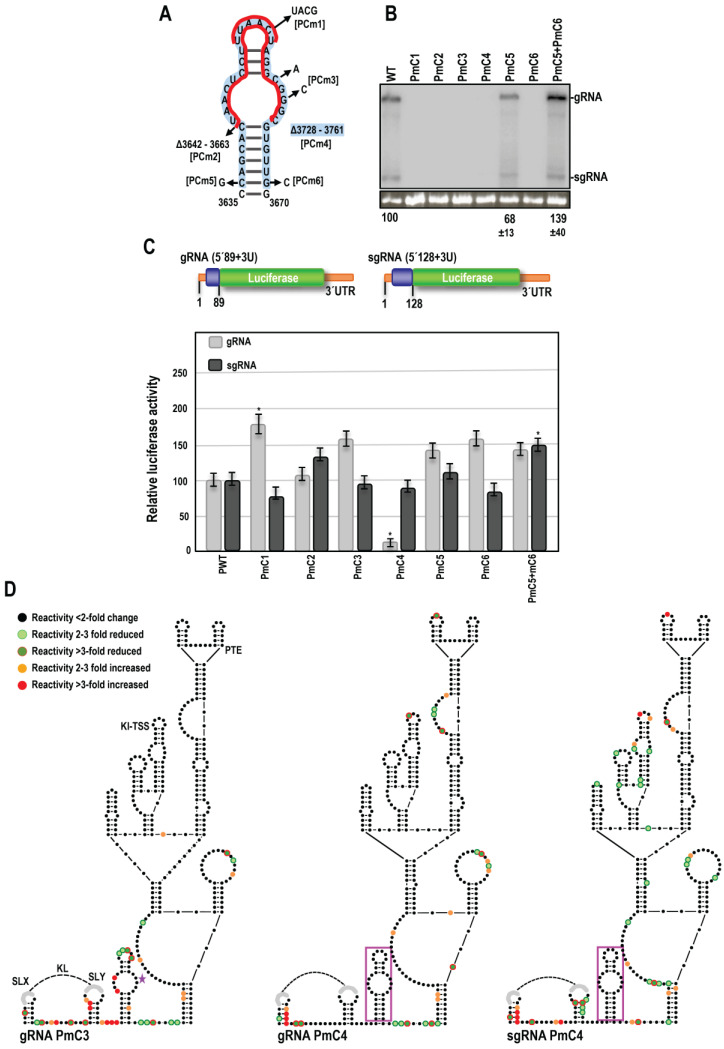
PEMV2 CAS is important for virus accumulation and efficient translation of the gRNA reporter construct. (**A**). Locations of mutations in the CAS hairpin. Names of mutations are bracketed. Red lines indicate some of the nucleotides that were altered or deleted. Blue shading denotes residues deleted in PmC4. (**B**). RNA gel blot analysis of total RNA extracted from systemically infected *N. benthamiana* leaves following agroinfiltration with either PEMV2 WT or derived CAS mutants. Ethidium bromide-stained 26S rRNA was used as a loading control for normalizing band intensities. (**C**). PEMV2 reporter constructs containing complete 3′UTR and 5′ end non-coding (orange) and coding (blue) sequences as previously described [27]. Relative luciferase activity of constructs was assayed in protoplasts. Error bars denote standard deviation for three independent assays. ‘*’ Denotes statistical difference with *p* < 0.05 analyzed using the *t*-test. (**D**). SHAPE RNA structure probing of PEMV2 gRNA and sgRNA CAS mutants. Dot colors represent changes in nucleotide reactivity in the mutant construct relative to WT (see legend to Figure 5). Star indicates position of the mutation.

To examine if CAS alternations in other umbraviruses lead to similar effects on virus accumulation, translation, and structural alterations in the 3′CITE region, the CAS of PEMV2 with its upstream KL element and downstream PTE/Kl-TSS 3′CITEs was also investigated. Figure 8A shows that full-length gRNA and sgRNA transcripts have similar SHAPE structure profiles in this region. However, unlike the SHAPE data for the OPMV CAS, the PEMV2 SHAPE data were consistent with the presence of the CAS structure in both gRNA and sgRNA transcripts (Figure 9B). Additionally, unlike OPMV, SLX was not supported in the gRNA transcripts, and neither SLX nor SLY were supported in the sgRNA transcripts. These data suggest that different umbravirus transcripts may assume different conformations in this region.

### 3.9. CAS Is Required for Efficient Accumulation of PEMV2 RNA In Vivo

Mutations similar to those used to investigate the OPMV CAS were introduced into PEMV2 CAS, and the effect on viral accumulation was similarly assessed by RNA gel blot analysis following A. *tumefaciens*-mediated infiltration of expression constructs in *N. benthamiana* (Figure 9A). Similar to OPMV CAS, converting the apical loop sequence into a UNCG tetraloop (PmC1), the deletion of the upper portion of CAS (PmC2), the alteration of residues in the internal loop (PmC3), or complete CAS deletion (PmC4) all reduced PEMV2 accumulation below the level of detection (Figure 9B). Single residue changes near the base of the stem (PmC5 and PmC6) reduced accumulation to 68% and below the level of detection, respectively, and similar to OPMV, the compensatory mutations together enhanced accumulation comparable to WT levels. These results confirm an important function for CAS in umbraviruses with different 3′CITEs.

### 3.10. PEMV2 CAS Is Required for Efficient Translation of gRNA but Not sgRNA Reporter Constructs In Vivo

The same mutations used to study the effects of OPMV CAS on translation were introduced into PEMV2 gRNA and sgRNA luciferase constructs (Figure 9C) [27]. Both constructs contained the complete 703 nt PEMV2 3′UTR, with the gRNA construct containing the 5′ 89 nt of the gRNA (5′89 + U) and the sgRNA construct containing the 5′128 nt of the sgRNA (5′128 + U) to accommodate the Kl-TSS long-distance RNA:RNA interacting sequences. Similar to OPMV, PmC1 increased luciferase activity of the gRNA construct by 1.7-fold relative to WT and did not significantly affect sgRNA luciferase activity, suggesting a possible related effect of replacing the apical loop sequence with a stable tetraloop (Figure 9C). In contrast with OPMV, PmC2 and PmC3 did not significantly affect translation of either the gRNA or sgRNA reporter constructs. The deletion of CAS (PmC4) decreased gRNA luciferase activity five-fold relative to WT, but unlike OPMV, did not similarly affect translation of the sgRNA construct. PmC5, PmC6, and PmC5 + PmC6 had little effect on translation of the PEMV2 gRNA and sgRNA constructs, unlike the differential effects of the analogous OPMV mutations. These results suggest that, with the exception of the apical loop alteration, PEMV2 CAS is functioning differently from OPMV CAS in supporting and repressing translation of gRNA and sgRNA constructs.

### 3.11. PEMV2 CAS Mutations Affect the Structure of Upstream Sequences

To determine the effects of CAS mutations on the structure of the PEMV2 3′CITEs and upstream KL element, full-length gRNA transcripts containing PmC3 and PmC4 were analyzed by SHAPE structure probing (Figure 9D). Unlike OPMV mC3, PmC3 caused significant reactivity changes in CAS residues and residues in surrounding regions, including (1) enhanced reactivity in the SLX and SLY stems; (2) enhanced and reduced residue reactivity between SLX and SLY; (3) substantially enhanced reactivity of the conserved “GAUC” adjacent to CAS; and (4) reactivity differences throughout CAS, including reduced reactivity of five of seven residues in the apical loop; and (5) reduced reactivity of the sequence between CAS and the 3′CITE structure. Similar to OPMV CAS mC3, only scattered differences were found within the downstream 3′CITE structure, including two consecutive residues in the base stem, and none within the Kl-TSS and PTE 3′CITEs. The deletion of CAS (PmC4) also caused a number of SHAPE reactivity changes both upstream and downstream of the deletion. The most prominent reactivity changes were enhanced reactivity in SLX stem residues, and similar to PmC3, reduced the reactivity of residues between the CAS and the 3′CITE structure and enhanced reactivity of the same two residues in the base stem of the 3′CITE structure (Figure 9D). The deletion of CAS also caused two consecutive residues to lose SHAPE reactivity in the bulge loop of the PTE, a critical region that forms a required pseudoknot within the CITE [22]. Since deletion of CAS yielded very different outcomes for translation of the gRNA and sgRNA reporter constructs, sgRNA PmC4 transcripts were also subjected to SHAPE probing. Whereas the CAS deletion in the sgRNA caused similar changes in SLX and in the sequence between the CAS and the 3′CITE structure as found for the gRNA, a substantial number of differences were present, including the critical PTE bulge region, likely due to the existence of additional sequence and/or different structures outside of this region between the gRNA and sgRNA transcripts. These results strongly suggest that PEMV2 CAS, unlike OPMV CAS, has a direct or indirect structural connection with the upstream KL element and sequences between the CAS and the 3′CITE structure. Overall, our results support CAS as a critical new translation element in umbraviruses, whose overall mode of action likely differs depending on the virus and whether the template is gRNA or sgRNA.

## 4. Discussion

Many (+)RNA plant viruses follow unconventional translational pathways often mediated by 3′CITEs present near the 3′ end of the viral genomes [4,5]. Umbraviruses are unusual in having extended 3′ UTRs that contain multiple 3′CITEs with different usages for translation of gRNA and sgRNA [17,30]. In this report, we identified an additional critical umbravirus element, the CAS, located just upstream of all centrally located umbravirus 3′CITEs. All CAS share conserved features, including highly conserved residues in their apical loops and at the base of the stem extending into flanking sequences, along with an internal bulge loop that can be symmetrical or asymmetrical. The 3′CITEs just downstream of the CAS are predicted to be perched atop extended supporting stems (with the exception of PiUV), placing CAS a short distance upstream from the base of all 3′CITE-supporting stems (Figure 2 and Figure 3). The positioning of 3′CITEs on extended stems may be advantageous for connecting the CITE to the 5′ end by long-distance RNA:RNA interactions, which are usually required for translation enhancer activity [4,5,43].

**Figure 10:**
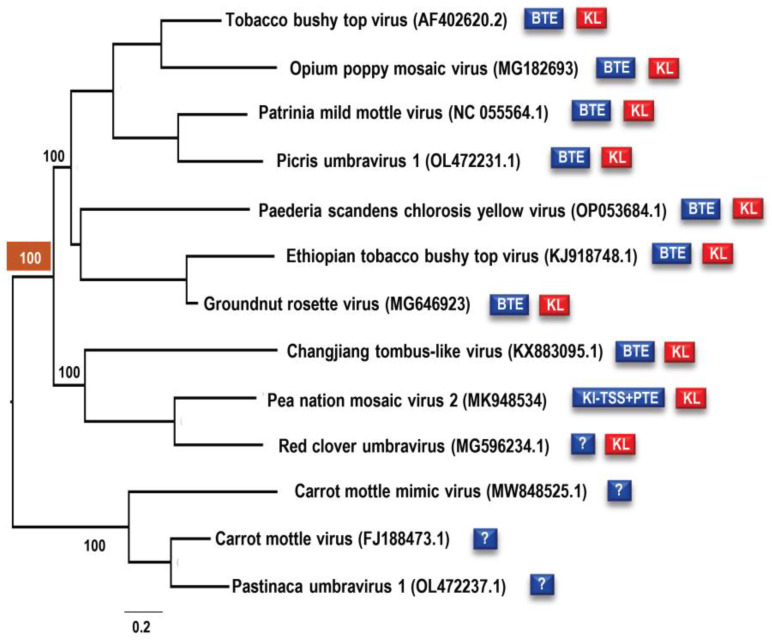
Maximum likelihood phylogenetic tree of umbraviruses based on 3′ UTR nucleotide sequences. Values above each branch indicate bootstrap support in the percentage out of 1000 bootstraps. The bottom scale bar indicates nucleotide substitutions per site. The blue and red boxes next to virus names indicate the type of CITE (if known) and the presence of the KL element. The bootstrap support for the branch containing closely related umbraviruses possessing the conserved KL element is highlighted in brown.

Eleven of the fourteen CAS have an additional element located 0 to 1 nt upstream from the conserved CAS flanking sequence, which consists of simple hairpins SLX and SLY with apical loops connected by a putative KL of three to five residues (Figure 1C). Unlike CAS, there is no conservation of sequences among these KL elements, although several KL contain the common umbravirus and carmovirus tertiary interaction sequence “GCCA”/”UGGC” [4]. In addition, umbraviruses that are more closely related, e.g., TBTV, OPMV, Changjiang3, PEMV2, and RCUV (Figure 10), have similar or identical loop sequences with several co-variant residues in the stems and poorly conserved sequences between the hairpins, suggesting a greater importance for maintaining the loop sequence. The disruption of the KL interaction in OPMV had no discernable effect on virus accumulation in *N. benthamiana* (Figure 7), suggesting that the OPMV KL element has a function that does not impact virus accumulation in single cells. While this report identifies the CAS as an important element for umbravirus accumulation, sequences further upstream of the KL element likely also play important roles. For umbravirus TBTV, alterations in a sequence upstream of the KL element decreased translation of ORF 1, which correlated with a decrease in viral accumulation and structural changes in BTE apical loops [44].

Many residues in both OPMV and PEMV2 CAS were critical for virus accumulation in *N. benthamiana* (Figure 5C and Figure 9C). For both OPMV and PEMV2, deleting the entire CAS hairpin (mC4 and PmC4, respectively) significantly reduced translation of gRNA and sgRNA2 OPMV reporter constructs and also significantly reduced translation of the PEMV2 gRNA construct. While these results strongly suggest an important role for CAS in translation, leading to the limited accumulation of mutant virus in *N. benthamiana*, an additional role for CAS in replication cannot currently be ruled out. Curiously, changing the conserved apical loop to a highly stable UNCG tetraloop [45] enhanced translation of gRNA but not sgRNA constructs (Figure 5C and Figure 9C), suggesting a conserved effect of this alteration. OPMV deletion mC2, which removed the apical loop among other sequences, enhanced translation of the sgRNA but not the gRNA, and the comparable deletion in PEMV2 constructs had no significant effect on translation. One possibility for these conflicting results is if the UACG apical loop assisted in gRNA CAS function by stabilizing the hairpin, thus inadvertently supporting translation of the gRNA reporter constructs.

Surprisingly, alterations and deletion of OPMV and PEMV2 CAS affected the SHAPE reactivity of only a few residues in the downstream 3′CITEs (Figure 5D and Figure 9D). For the OPMV BTE, mC1 and mC4 reduced the flexibility of residues at the 5′ base and residues just downstream from the three apical hairpins. This latter location is within a 4-nt segment important for eIF4G binding [11]. A model for how BYDV BTE functions was recently proposed, suggesting that eIF3 interacts simultaneously with both the BTE (through binding to eIF4G) and the 5′ UTR to stabilize the long-distance interaction [14,15,46]. eIF3 also enhanced the recruitment of 40S ribosomal subunits to the BTE-associated eIF complex and mediated the transfer of the 43S complex to the 5′ end [15]. One possible function for the OPMV CAS could be maintaining the eIF4G-interacting residues in a conformation that allows for eIF4G binding. This suggestion, however, does not explain why mC1 had a positive effect on translation of the gRNA construct. Interestingly, the deletion of PEMV2 CAS (PmC4), the only alteration that significantly decreased translation of the gRNA luciferase construct, decreased the flexibility of two adjacent PTE residues in the large G-rich bulge loop (Figure 9D). These residues are known to form a pseudoknot with a C-rich sequence located between the two apical hairpins, which is important for the binding of eIF4E to PTE [21,22].

OPMV and PEMV2 gRNA transcripts differed in whether SHAPE data supported the CAS and KL element structures. For OPMV, the 3′CITE and KL element structures were consistent with the SHAPE data, but neither the gRNA nor sgRNA SHAPE profile supported the CAS structure. In contrast, PEMV2 gRNA and sgRNA transcript SHAPE data supported their CAS structures but not the structures of SLX (gRNA) or SLX and SLY (sgRNA). Since these hairpins are phylogenetically conserved, and since compensatory mutations at the base of the CAS stem restored gRNA levels in vivo for both OPMV (Figure 5B) and PEMV2 (Figure 9B), the lack of support for CAS from SHAPE probing of OPMV transcripts could reflect structural plasticity of the RNA in this region. Alternatively, the lack of SHAPE support for the structures could be an unintended consequence of artificially folded transcripts (i.e., transcripts did not fold co-transcriptionally, as would occur in vivo [47]). Interestingly, the SHAPE data also did not support the structure of a highly conserved hairpin just upstream from the PEMV2 3′TSS [48] that was confirmed to exist by compensatory mutagenesis, suggesting that different conformation of the 3′ UTR may be present during the virus infection cycle, as was found for related carmoviruses [25,49].

A search for hairpins with CAS-conserved features upstream of 3′CITEs in virus members of other genera in the *Tombusviridae* (e.g., BYDV) did not reveal the existence of hairpins with similar conserved features, suggesting that CAS may be uniquely associated with umbraviruses. Other hairpins upstream of 3′-proximal CITEs have been found in betanecroviruses TNV-D and beet black scorch virus [28], but their function remains enigmatic. Mutations in one hairpin in TNV-D reduced viral translation in wheat germ extracts but had no effect on the accumulation in protoplasts, while similar mutations in a second hairpin reduced accumulation in protoplasts but had no significant effect on in vitro translation. TNV-A has a short segment near the start of its coat protein ORF that was also critical for both virus accumulation and translation of reporter constructs. However, unlike the CAS, this segment is distal to its 3′ UTR-located BTE 3′CITE [50].

Umbraviruses whose interior 3′CITEs have not yet been identified (CMoMV, CMoV, PasUV1, and RCUV) are predicted to have similar structured elements atop extended stems that likely constitute novel CITE classes (Figure 3). All umbravirus 3′CITEs (known and unknown) are positioned at a similar distance downstream from CAS hairpins and upstream of newly identified replication elements (“Trio”) that are completely conserved in five umbraviruses (including OPMV and PEMV2) and partially conserved in most of the remaining viruses [48]. This identical positioning of different umbravirus 3′CITEs between conserved elements suggests that they are modular units inserted by RNA recombination into an existing receptive location flanked by CAS and Trio hairpins. The different effects of CAS mutations on translation of luciferase constructs and on the structure of the upstream KL elements and downstream 3′CITEs suggest independent evolution has optimized CAS functions according to the virus and the host environment. Carmoviruses, which share a close evolutionary relationship with umbraviruses, also contain different CITEs (TSS/PTE/TED/ISS) between conserved 3′ ends and the coat protein ORF [9,23,51,52]. These examples invoke the framework of modular evolution that was proposed for members of the *Tombusviridae* [39,53] and was demonstrated for melon necrotic spot virus (MNSV) [54], which gained a second 3′CITE through recombination with an unrelated virus, allowing MNSV to bypass host resistance associated with the original CITE.

## Data Availability

Not applicable.
